# Associations of serum 25(OH)D levels with physical performance and anabolic hormones in young men

**DOI:** 10.3389/fphys.2023.1049503

**Published:** 2023-02-07

**Authors:** Akseli Laaksi, Ilkka Laaksi, Harri Pihlajamäki, Jani P. Vaara, Tiina Luukkaala, Heikki Kyröläinen

**Affiliations:** ^1^ Faculty of Medicine and Health Technology, University of Tampere, Tampere, Finland; ^2^ Centre for Military Medicine, Finnish Defence Forces, Riihimäki, Finland; ^3^ Department of Orthopedics and Traumatology, Seinäjoki Central Hospital, Seinäjoki, Finland; ^4^ Department of Leadership and Military Pedagogy, National Defence University, Helsinki, Finland; ^5^ Research, Development and Innovation Centre, Tampere University Hospital, Tampere, Finland; ^6^ Health Sciences, Faculty of Social Sciences, University of Tampere, Tampere, Finland; ^7^ Faculty of Sport and Health Sciences, University of Jyväskylä, Jyväskylä, Finland

**Keywords:** vitamin D, young men, anabolic hormones, physical performance, military

## Abstract

**Purpose:** The present study examined the association of vitamin D measured by serum 25(OH)D with physical performance outcomes and serum levels of anabolic hormones in young men.

**Methods:** 412 young men (age 19 ± 1 year) entering their compulsory military service volunteered to participate in the study. The study consisted of two groups from two different military bases: Group A was studied in January and group B in July. The groups were first compared with each other and due to statistically significant (*p* < 0.001 analyzed with independent samples *t*-test) differences in physical condition (sit-up, push-up, and standing long jump-tests and testosterone levels) between the groups, groups were analyzed separately. The serum levels of 25(OH)D, testosterone (TES), sex hormone binding globulin (SHBG), and insulin-like growth factor-1 (IGF-1) were analyzed by electrochemiluminescence immunoassay. Physical performance tests consisted of muscular fitness (sit-ups, push-ups, standing long jump) and aerobic fitness (12-minute-running) tests. The association of serum 25(OH)D with physical performance tests and anabolic hormones was analyzed using linear regression.

**Results:** After controlling for the group, body mass index, and leisure-time physical activity, serum 25(OH)D level was positively associated with aerobic and muscular fitness (*β* = 0.15–0.20, all *p* < 0.05). Also, the participants with sufficient serum 25(OH)D levels (≥75 nmol/L) had better aerobic and muscular fitness and higher TES in group B, and better upper extremity muscular fitness in group A (all *p* < 0.05). In group A, there were 166 participants with serum levels of 25(OH) D < 75 nmol/L and 18 ≥ 75 nmol/L. In group B, the amounts were 92 (<75 nmol/L) and 136 (≥75 nmol/L), respectively.

**Conclusion:** Serum 25(OH)D was positively associated with both aerobic and muscular fitness and those with sufficient vitamin D levels, had higher levels of TES. Thus, maintaining a sufficient serum 25(OH)D level may be beneficial for physical performance and anabolic state in young men.

## 1 Introduction

Vitamin D is primarily produced endogenously in the skin from 7-dehydrocholesterol, which is induced by ultraviolet B radiation (UVB) from the sun. In addition to endogenous production, vitamin D can be obtained from the diet, such as fatty fish, fortified dairy products, and dietary supplements. Vitamin D is metabolized in the body by hydroxylation in the liver to 25(OH)D, which is then transported to the kidneys or target tissues to be hydroxylated again to its most active form of 1,25(OH)_2_D (Holick, 2007). After the second hydroxylation vitamin D mediates its effects through a specific receptor in cells called vitamin D receptor (VDR) (Haussler et al., 1998). Vitamin D status is assessed by serum 25(OH)D levels (Utiger, 1998). The Nordic Nutrition Recommendations set 50 nmol/L in serum 25(OH)D as a lower limit value for vitamin D sufficiency (Nordic Council of Ministers, 2014). However, the Global Endocrine Society’s Practice Guidelines Committee recommends that a serum 25(OH)D level of ≥75 nmol/L is considered sufficient for musculoskeletal health (Holick, 2017).

Vitamin D is known to have an important role in bone metabolism by promoting intestinal calcium and phosphate absorption, renal tubular calcium reabsorption, and calcium mobilization from the bone by its most active form of 1,25(OH)_2_D (Charoenngam et al., 2019). However, it is not so well-known that vitamin D may also be associated with physical performance. In cross-sectional studies among healthy adults and athletes, vitamin D has been found to be positively associated with muscular power and strength (Hamilton et al., 2014; Koundourakis et al., 2014; Hildebrand et al., 2016) and aerobic performance (Koundourakis et al., 2014; Carswell et al., 2018; Zeitler et al., 2018). Nevertheless, a narrative review showed that the association between vitamin D and physical performance in athletes has been inconclusive (Ksiażek et al., 2019), while there exists only a limited number of studies among healthy adults.

The beneficial effects of vitamin D on physical performance may exist through increasing blood levels of anabolic hormones such as testosterone (TES) and insulin-like growth factor-I (IGF-1). A large (*n* = 20 567) systematic review and meta-analysis showed an association between vitamin D deficiency and decreased testosterone levels (D’Andrea et al., 2021). A positive correlation between serum 25(OH)D level and IGF-1 has been found in several observational studies as well (Forouhi et al., 2008; Bogazzi et al., 2011; Ameri et al., 2013a). However, studies that have examined the association of these hormones with serum 25(OH)D together with physical performance are rare. In addition to TES level, it is important to measure sex hormone binding globulin (SHBG) as the circulating TES is mostly bound on albumin and SHBG, and thus high SHBG could indicate lower bioavailable TES (Goldman et al., 2017).

Therefore, the present cross-sectional study aimed to examine whether there is an association between 25(OH)D levels, physical performance (aerobic and muscular fitness), and anabolic hormones in young men. It was hypothesized that serum 25(OH)D levels would have positive associations with physical performance and levels of anabolic hormones.

## 2 Materials and methods

### 2.1 Participants

Young men (*n* = 412; age 19 ± 1 year, height 179 ± 7 cm, body mass 75 ± 13 kg, body mass index 24.0 ± 3.7), performing their compulsory military service, volunteered to participate in the present study. Written informed consent was given to the participants after passing the military entry medical examination as healthy. The present study has been approved by the Ethics Committee of the Pirkanmaa Hospital District, ETL code R17155, HUS/1557/2018, and the Finnish Defence Forces (AN21508, AP10027, AQ24718, AR13336).

#### 2.2 Study protocol

The present cross-sectional study had two separate groups: group A (*n* = 184) and group B (*n* = 228) in two different military units in Finland. Groups were compared and then analyzed separately due to differences in physical performance; the participants in group A were conducting their military service in rapid deployment forces that requires better physical condition than a standard military service in Finland. The physical performance tests were arranged during the first 2 weeks of military service. In group A, the blood samples were drawn in the winter (January), while in another location (group B), the blood samples were drawn in the summer (July). In northern latitude countries, such as Finland, an adequate amount of sunlight exposure for vitamin D production in the skin is possible only between March and October (Engelsen et al., 2005). In addition, levels of serum TES, SHBG, and IGF-1 were analyzed for evaluating the anabolic status of the participants. The physical performance was studied by conducting sit-up, push-up, standing long jump, and 12-min running tests. The level of ≥75 nmol/L of serum 25(OH)D was used as sufficient in the present study. Group, body mass index (BMI), and leisure-time activity were used as covariates. Group was used as a covariate because the groups were measured during different seasons (summer/winter) and therefore, this difference can affect vitamin D levels through differences in sunlight exposure and further because there were fundamental differences in physical performance between the groups. BMI was selected due to its effects on serum 25(OH)D levels and physical performance. Leisure-time physical activity was selected for its effect on physical performance and the possible effect on serum 25(OH)D levels if the participants spend a lot of time outdoors while exercising and are thus more exposed to the sun.

### 2.3 Blood analyzes

After the venous blood samples were obtained from the antecubital vein, the samples were centrifuged at 2000 g for 20 min at room temperature for serum separation. Serum samples were then frozen and stored at −20°C for later analysis. The measurements of serum 25(OH)D were performed using electrochemiluminescence immunoassays (ECLIA). Levels of TES and SHBG were measured by electrochemiluminescence immunoassay (Immulite 100; Siemens Healthcare Diagnostics Products Ltd., Gwynedd, United Kingdom). IGF-1 was analyzed by electrochemiluminescence immunoassay (Siemens Immulite 2000 XPI, Siemens Healthcare Diagnostics Products Ltd., Gwynedd, United Kingdom). The inter-assay coefficients of variance (CV) for assays of TES, SHBG, and IGF-I were 7.0%–7.2%, 4.5%–6.2%, and 3.7%–7.4%, and that of sensitivity 0.5 nmol/L, 0.02 nmol/L, and 2.6 nmol/L, respectively.

### 2.4 Physical performance

Aerobic performance was measured by a 12-min running test, an explosive power output of the lower limbs by a standing long jump test, and dynamic muscular endurance by 1-min push-up and sit-up tests. In the 12-min running test, participants try to run as far as possible in the given time. The results can be used to estimate the maximum oxygen uptake (VO_2_ max) of the participants by using the formula: $VO2\max=\left(d-504.9\right)÷44.73$ (d = the distance covered in the given time) (Cooper, 1968). In a systematic literature review and meta-analysis, where different running tests were compared, the 12-min running test was shown to be relatively accurate to estimate VO_2_ max when a laboratory test is not possible (Mayorga-Vega et al., 2016). Cooper (1968) demonstrated that the correlation coefficient between a directly measured VO_2_ max and a 12-min running test is 0.86. In addition, the reliability coefficient has been reported to be 0.96, respectively (Penry et al., 2011).

The purpose of the standing long jump is to evaluate the maximum and explosive power output of the lower limbs. In the present study, the standing long jump was performed three times, starting on the ground level with the legs close to each other. Explosive bilateral takeoff was assisted by powerful swinging of the upper body and arms. The landing of each jump was performed bilaterally. The result of the best jump was expressed in meters as the shortest distance from the landing to the starting line, and the best result was taken for further analysis. Adequate recovery time (2–3 min) between performances was used. Standing long jump test reliability coefficients have been shown to vary between 0.93 and 0.96 (Markovic et al., 2004).

Dynamic muscular endurance of the abdominal and hip flexor muscles was measured by performing the sit-ups for as many repetitions as possible in 60 s. Sit-ups were done with each participant lying on the floor with hands behind his neck and directing his elbows forward. The knees were flexed at an angle of 90 deg with the legs slightly abducted. During the movement, each participant lifted his upper body and touched his elbows to his knees. The partner supports the performer’s ankles, following the correct performance technique and counting the number of repetitions (Viljanen et al., 1991).

The dynamic strength and endurance of the muscles of the shoulder area and upper extremities, as well as the static endurance of the muscles supporting the movement, are assessed by performing as many push-ups as possible in 60 s. Push-ups were started from the lowest face-down position. Each participant’s hands were kept at shoulder-width and level. The fingers were directed forward, and the legs were kept parallel and close to each other. During the movement, the arms were fully extended, and the torso was straightly tensed. In the second phase, the torso was lowered down to an elbow angle of 90°. The result of this test was expressed as the number of push-ups completed in 60 s [ACSM’s Guidelines for Exercise Testing and Prescription, 2000 (ACSM, 2000)]. The reliabilities for both the sit-up and the push-up tests have been reported to be high (intraclass correlation values ranged from 0.92 to 0.95) (Ryman Augustsson et al., 2009).

The results of the push-up, sit-up, and standing long jump tests were transformed into z-scores by standardizing them to be co-dimensional with each other. Then the mean of each z-score formed a common continuous variable, called muscular fitness index (MFI), as reported previously (Vaara et al., 2014).

Leisure-time physical activity was studied from self-fillable questionnaires. Participants responded to questions “How many times a week on average do you do endurance-type exercise?” and “How many times a week on average do you engage in exercise that develops muscular strength (e.g., gym training)?” (Think about the last 3 months) with eight response categories: 1 = none; 2 = once a week; 3 = 2 times a week; 4 = 3 times a week; 5 = 4 times a week; 6 = 5 times a week; 7 = 6 times a week; 8 = 7 times or more per week. In general, regarding physical activity questionnaires, acceptable to good reliability but poor to moderate validity has been reported (Helmerhorst et al., 2012).

### 2.5 Statistical analysis

Bivariate Pearson’s correlations (r) between serum 25(OH)D and physical performance, anabolic hormone levels, and controlling covariates were performed to show the logical progression of statistical choices and to confirm the relevance of candidate covariates. Then linear regression was used to assess the association of serum 25(OH)D levels with physical performance and anabolic hormone after controlling for the group, BMI, and leisure-time physical activity. The size of the analyzed data in multivariable-adjusted analyses varied from n = 329 to *n* = 349 due to some missing data in variables of BMI and leisure-time physical activity (*n* = 24), and physical performance and anabolic hormones variables themselves (see Table 2). Additionally, the groups were analyzed separately by dividing them into subgroups using a sufficient level of serum 25(OH)D ≥ 75 nmol/L (Holick, 2017) as a limit value. Differences in outcome variables between the subgroups were analyzed using independent samples t-tests. The results of the t-tests have been reported according to whether the equivalence of the variances has been assumed or not. Statistical analyses were performed with IBM SPSS Statistics for Macintosh, Version 27.0. Armonk, NY: IBM Corp (released 2020). A two-sided *p*-value under 0.05 was considered statistically significant. Post-hoc power analyses were performed using G*Power version 3.1.9.6.

## 3 Results

The mean serum 25(OH)D levels were significantly lower in group A (data gathered in January) than in group B (data gathered in July) (56.3 ± 14.5 nmol/L vs 78.3 ± 18.5 nmol/L, *p* < 0.001). The mean serum 25(OH)D of the whole study population was 68 ± 20 nmol/L. The results of physical performance and anabolic hormones are presented in Table 1.

**TABLE 1:** Physical performance and anabolic hormone status and covariates between group A and B (N = 412). Standing long jump (SLJ), Muscular fitness index (MFI); Testosterone (TES); Sex hormone binding globulin (SHBG); Insulin-like growth factor-1 (IGF-1), Body mass index (BMI). Differences between groups were tested using Independent Samples *t*-test.

	Group A			Group B			
	*n*	Mean	(Sd)	*n*	Mean	(Sd)	*p*-value
25(OH)D (nmol/L)	184	56.3	(14.5)	228	78.3	(18.5)	<0.001
*Physical performance*							
Push-ups (reps/min)	178	39	(16)	207	26	(12)	<0.001
Sit-ups (reps/min)	178	42	(10)	208	35	9)	<0.001
SLJ (cm)	178	230	(30)	208	220	(30)	0.004
12-min-run test m)	176	2420	(334)	203	2280	(337)	<0.001
*Anabolic hormones*							
TES (nmol/L)	184	20.1	(7.5)	228	11.5	(3.7)	<0.001
SHBG (nmol/L)	131	34.1	(13.7)	228	32.6	(11.6)	0.284
IGF1 (nmol/L)	184	25.1	(6.4)	228	29.6	(6.8)	<0.001
Covariates							
BMI	204	24.2	(3.3)	221	23.1	(3.9)	0.003
Leisure-time physical activity (times/week)	183	2.4	(1.6)	226	1.6	(1.5)	<0.001

In the whole population, serum 25(OH)D levels correlated statistically significantly with testosterone (r = −0.294) and IGF-1 (r = 0.231); *p* < 0.001, *n* = 412 for both. When groups were analyzed separately, there were no statistically significant Pearson correlations (r) between serum 25(OH)D and physical performance or anabolic hormones in winter group A. Instead, in summer group B, positive weak correlations (all *p* < 0.05) were found between 25(OH)D and push-ups (r = 0.208), sit-ups (r = 0.235), standing long jumps (r = 0.257), 12-min running test (r = 0.288), MFI (r = 0.269), TES (r = 0.157) and SHBG (r = 0.138).

After controlling the whole study population for the group, body composition, and leisure-time physical activity, 25(OH)D level was positively associated with push-up, sit-up, standing long jump, and 12-min running tests, and with MFI (Table 2).

**TABLE 2:** Associations of serum 25(OH)D physical performance, and anabolic hormones using linear regression analysis adjusting analysis by group, body mass index, and exercise activity. Differences between groups were tested using Linear Regression analysis. Results are shown using unstandardized beta (Unstand. *β*) with lower (Lower L) and upper limit (Upper L) of 95% confidence interval, standardized beta (Stand. *β*), and *p*-values. Push-ups in 1 minute, Sit-ups in 1 min, SLJ = standing long jump, MFI = muscular fitness index (push-up, sit-up, and SLJ combined), 12 MR = 12-min running test, TES = testosterone, SHBG = sex hormone binding globulin, IGF-1 = insulin-like growth factor 1. The limit of a statistically significant (*p* < 0.05) t-value is t>|1.960| and highly significant (*p* < 0.001) the limit is t>|3.291|.

	Physical performance					Anabolic hormones		
	Push-ups	Sit-ups	SLJ	MFI	12 MR	TES	SHBG	IGF-1
	*n* = 330	*n* = 331	*n* = 331	*n* = 331	*n* = 349	*n* = 339	*n* = 329	*n* = 339
25(OH)D								
Unstand. *β*	0.121	0.089	0.003	0.026	2.696	0.012	0.051	0.021
Lower L	0.038	0.033	0.001	0.012	0.780	−0.021	−0.026	−0.021
Upper L	0.205	0.146	0.004	0.040	4.613	0.045	0.128	0.064
Stand. *β*	0.161	0.175	0.171	0.198	0.155	0.035	0.078	0.061
t-value	2.860	3.122	2.930	3.601	2.767	0.719	1.294	0.989
*p*-value	0.005	0.002	0.004	<0.001	0.006	0.473	0.197	0.324
t-values for								
BMI	−4.301	−6.405	−6.838	−7.160	−8.517	−4.774	−5.920	1.088
Activity	3.087	2.662	1.088	2.714	2.235	−0.522	−0.435	−0.136
Group	−8.032	−7.022	−3.796	−7.530	−5.621	−13.536	−2.283	3.755

Of controlling variables, BMI had a weak negative correlation with serum 25(OH)D (r = −0.140) and physical performance tests (sit-ups: r = −0.234, standing long jumps: r = −0.314, 12-min running test: r = −0.249 and MFI −0.249). Leisure-time physical activity did not correlate with serum 25(OH)D but had weak positive correlations with all physical performance variables and TES (sit-ups: r = 0.259, push-ups: r = 0.282, standing long jumps: r = 0.172, 12-min running test: r = 0.165, MFI: r = 0.276 and TES: r = 0.120).

The differences between participants that had sufficient (≥75 nmol/L) and insufficient (<75 nmol/L) serum 25(OH)D levels were also tested with independent samples *t*-test (Table 3). As the physical condition differed between groups A and B (*p* < 0.001), due to differences in military units, the groups were analyzed separately. In group B, the participants with serum 25(OH)D level higher than the sufficient level, had significantly better results in the 12-min run test (*p* < 0.001), sit-ups (*p* = 0.048), standing long jumps (*p* = 0.010), and muscular fitness index (0.031) and higher levels of TES (0.004), while in group A, there were only significant differences in push-ups (*p* = 0.022), respectively.

**TABLE 3:** Differences of the mean (±SD) anabolic hormone levels and physical performance test results between the subgroups divided by sufficient level (75 nmol/L) of serum 25 (OH) D in group A (data gathered in January) and B (data gathered in July). Standing long jump (SLJ); muscular fitness index (MFI); testosterone (TES); sex hormone binding globulin (SHBG); testosterone-sex hormone binding globulin ratio (TES/SHBG); insulin-like globulin factor-1 (IGF-1). Differences between groups were tested using Independent Samples *t*-test.

	Group A serum 25(OH)D (nmol/L)			Group B serum 25(OH)D (nmol/L)		
	<75.0	≥75.0		<75.0	≥75.0	
	Mean (SD)	Mean (SD)	*p*-value	Mean (SD)	Mean (SD)	*p*-value
25(OH)D	53.5 (11.7) *n* = 166	82.7 (11.0) *n* = 18		61.2 (9.9) *n* = 92	89.8 (13.3) *n* = 136	
*Physical performance*						
Sit-ups (reps/min)	42 (10) *n* = 160	45 (8.7) *n* = 18	0.128	34 9) *n* = 84	37 (10) *n* = 124	0.033
Push-ups (reps/min)	38 (16) *n* = 160	48 (16) *n* = 18	0.010	25 (11) *n* = 84	27 (13) *n* = 112	0.192
SLJ (cm)	231 (30) *n* = 160	238 (28) *n* = 18	0.358	216 (27) *n* = 84	227 (30) *n* = 124	0.007
MFI	−0.24 (2.51) *n* = 160	1.01 (2.45) *n* = 18	0.044	−2.36 (2.21) *n* = 84	−1.55 (2.44) *n* = 124	0.016
12-min run test m)	2400 (335) *n* = 158	2540 (313) *n* = 18	0.107	2190 (283) *n* = 82	2340 (357) *n* = 121	<0.001
*Anabolic hormones*						
TES (nmol/L)	20.1 (7.5) *n* = 166	19.7 (7.6) *n* = 18	0.816	10.6 (3.4) *n* = 92	12.1 (3.7) *n* = 136	0.002
SHBG (nmol/L)	33.9 (13.9) *n* = 114	35.8 (12.0) *n* = 17	0.591	31.3 (11.7) *n* = 92	33.5 (11.5) *n* = 136	0.172
IGF-1 (nmol/L)	25.0 (6.4) *n* = 166	26.0 (6.4) *n* = 18	0.525	29.1 (6.9) *n* = 92	29.9 (6.7) *n* = 136	0.408

Using effect sizes, derived from independent samples *t*-test, actual *post hoc* power showed to be at least 80%.

## 4 Discussion

The main finding of the present study demonstrate that 25(OH)D was positively associated with aerobic and muscular fitness test results after controlling for the group, body composition, and physical activity. In addition, in group B (data gathered in summer), the participants with serum 25(OH)D level higher than the sufficient level (≥75 nmol/L) had significantly better results in aerobic performance, muscular fitness, and higher levels of TES. In group A (data gathered in winter), only push-up test results differed statistically significantly between the subgroups based on sufficient serum 25(OH)D levels, respectively. The groups were analyzed separately due to fundamental differences in their physical condition. The participants in group A were conducting their military service in rapid deployment forces requiring better physical performance than group B, in which the participants were in standard military service. As group A was measured in winter when the vitamin D intake from the sunlight is lower, the association of vitamin D would not have been visible if the groups had been analyzed together.

Due to the compulsory military service in Finland, the participants in the present study represent the normal population of young men. Although most of the studies in this field have been conducted on athletes, the results coincide with the present study, showing positive relationships between serum 25(OH)D levels and muscular power and endurance (Hamilton et al., 2014; Koundourakis et al., 2014; Hildebrand et al., 2016; Heileson et al., 2022). The mechanisms of how vitamin D affects muscular fitness remain hypothetical, although there are several potential pathways. Vitamin D is known to influence both calcium and phosphate metabolism (Charoenngam et al., 2019). Calcium has a fundamental role in muscle contraction (Berchtold et al., 2000) and thus, vitamin D may have indirect beneficial effects on muscle contraction and strength *via* its effects on systemic calcium levels (Girgis et al., 2013). In the present study, serum 25(OH)D levels were associated with better results in tests requiring efficient muscle actions (push-ups, sit-ups, standing long jump), indicating that it could be enhanced by vitamin D. Also, vitamin D has been suggested to have direct effects on skeletal muscle through vitamin D receptor (VDR), which has been found in human skeletal muscle cells (Pojednic and Ceglia, 2014). VDR-mediated effects have been suggested to have rapid non-genomic and genomic effects on intracellular calcium and phosphate homeostasis, muscle differentiation, contractile protein expression/activity, and phospholipid composition (Girgis et al., 2013). This is supported by abnormal skeletal muscle development and reduced strength in VDR-knockout mice (Endo et al., 2003; Girgis et al., 2015). This could be associated with better progress in training muscular fitness for those with sufficient vitamin D levels. The Global Endocrine Society’s Practice Guidelines Committee’s recommendation for 25(OH)D level for maximum musculoskeletal health is ≥ 75 nmol/L (Holick, 2017). Interestingly, in group B, participants above that level had better outcomes in almost all variables in physical performance and higher TES. In group A, parallel results were found but only push-ups were statistically significant.

The present study also showed a positive relationship between serum 25(OH)D and aerobic performance. Associations between serum 25(OH)D and aerobic performance have previously been shown in several studies (Forney et al., 2014; Carswell et al., 2018; Zeitler et al., 2018). Notably, in line with the present study (Carswell et al., 2018), found in military recruits that serum 25(OH)D levels were associated with aerobic performance, although they reported a non-significant association with muscular strength and power. The association between serum 25(OH)D levels and VO_2_max could relate to VDRs as well. VDRs have also been found in cardiac muscle cells (Reddy Vanga et al., 2010), indicating that vitamin D may also have beneficial effects on aerobic performance by influencing oxygen transportation and utilization (Dahlquist et al., 2015).

In addition to the direct effects of vitamin D through VDR and the indirect effect on calcium homeostasis, vitamin D may have a beneficial impact on physical performance by increasing the levels of TES in the blood (Wehr et al., 2010; Nimptsch et al., 2012; Anic et al., 2016). VDRs have also been found in areas of the male reproductive tract such as the Leydig cells (Jensen, 2014). Testosterone is known to induce increased muscle growth, i.e., hypertrophy, by multiple mechanisms which include increasing the size and number of muscle fibers, increasing the number of myonuclei, and increasing the number of muscle satellite cells. This leads to increased muscular strength and mass (Kautiainen et al., 2002; Herbst and Bhasin, 2004; Kadi, 2008) Therefore, it can be concluded that higher vitamin D levels may indirectly improve muscular strength and thus physical performance. In the present study, TES was found higher in participants with sufficient (≥75 nmol/L) serum 25(OH)D levels in group B. Most of the circulating TES is bound to albumin and SHBG and higher SHBG levels indicates lower bioavailable TES levels and thus alter the effects of TES in the human body (Goldman et al., 2017). Nevertheless, there were no significant results on the SHBG levels in the present study (only a weak positive correlation in group B, which disappeared after adjusting for covariates). IGF-1, on the other hand, is a hormone that, among numerous other effects, regulates anabolic and catabolic pathways in skeletal muscle (Yoshida and Delafontaine, 2020). Vitamin D has been shown to increase serum IGF-1 levels through the induction of transcription of several genes in the liver (Ameri et al., 2013b) IGF-1 is also associated with better physical performance outcomes in some studies (Cappola et al., 2001; Nindl et al., 2011). A systematic review and meta-analysis showed a significant increase in IGF-1 levels in participants supplemented daily with ≤25ug of vitamin D. In the present study, there was a weak positive correlation between IGF-1 and serum 25(OH)D levels, but the effect disappeared after adjusting for seasonally presentative group effect.

The present study had several strengths. Muscular fitness was measured with three different muscle fitness tests: push-up, sit-up, and standing long jump which gives a comprehensive image of the muscular fitness state of the participants. Importantly, regression models were also controlled with BMI. High fat mass leads to decreased serum 25(OH)D levels (Wortsman et al., 2000) and impairs physical performance (Mattila et al., 2007) and hence is a major confounding factor. Another strength of the present study is the assessment of anabolic hormones TES and IGF-1. In addition, it is important to explore the seasonal variation of serum25(OH)D levels, especially, in high-latitude countries such as Finland, where adequate sunlight exposure exists only in a short period of summer months (Engelsen et al., 2005). Still, the study has some limitations. In group A, there were only 18 participants who reached over the sufficient serum 25(OH)D level of 75 nmol/L which may explain the lack of statistically significant differences between the sufficient and insufficient participants. The participants in group B had higher serum 25(OH)D levels and thus more statistical power in the sufficient group. The greatest limitation of the present study is that it is a cross-sectional study and therefore causal conclusion cannot be made. It is possible that the associations between serum 25(OH)D levels and physical performance could be partly explained by reverse causation: the more physically active participants may spend more time outdoors exposed to sunlight, hence have a better physical condition as well as higher serum 25(OH)D levels.

In conclusion, the hypotheses were supported, and the present study shows that serum 25(OH)D level could be positively associated with aerobic and muscular fitness. It is also possible that vitamin D is associated with physical performance only at a sufficient level of serum 25(OH)D, and the effects may be mediated or moderated through increasing the serum levels of testosterone. Further randomized control trial (RCT) research is needed in which the serum level of 25(OH)D must reach the sufficient limit for the supplementation group. This could be conducted in Finland during wintertime as we noticed that only a minor share of young Finnish men reaches the recommended 25(OH)D threshold in winter.

## Data Availability

The raw data supporting the conclusion of this article will be made available by the authors, without undue reservation.
